# Computer-based tools for assessing micro-longitudinal patterns of cognitive function in older adults

**DOI:** 10.1007/s11357-016-9934-x

**Published:** 2016-07-29

**Authors:** Laura J. E. Brown, Tim Adlam, Faustina Hwang, Hassan Khadra, Linda M. Maclean, Bridey Rudd, Tom Smith, Claire Timon, Elizabeth A. Williams, Arlene J. Astell

**Affiliations:** 1 School of Psychological Sciences and Manchester Centre for Health Psychology, The University of Manchester, Room S32, Second Floor, Zochonis Building, Brunswick Street, Manchester, M13 9PL UK; 2 Designability, Royal United Hospital, Bath, UK; 3 School of Systems Engineering, University of Reading, Reading, UK; 4 Oxford Technologies Limited, Oxford, UK; 5 Institute of Health and Wellbeing, University of Glasgow, Glasgow, UK; 6 School of Social and Health Sciences, University of Abertay, Dundee, UK; 7 Generic Robotics Ltd, Reading, UK; 8 Institute of Food and Health, University College Dublin, Dublin, Ireland; 9 Human Nutrition Unit, University of Sheffield, Sheffield, UK; 10 Centre for Assistive Technology and Connected Healthcare (CATCH), University of Sheffield, Sheffield, UK; 11 Ontario Shores Centre for Mental Health Sciences, Whitby, Canada

**Keywords:** Cognition, Repeated measures, Health, Assessment, Validation

## Abstract

Patterns of cognitive change over micro-longitudinal timescales (i.e., ranging from hours to days) are associated with a wide range of age-related health and functional outcomes. However, practical issues of conducting high-frequency assessments make investigations of micro-longitudinal cognition costly and burdensome to run. One way of addressing this is to develop cognitive assessments that can be performed by older adults, in their own homes, without a researcher being present. Here, we address the question of whether reliable and valid cognitive data can be collected over micro-longitudinal timescales using unsupervised cognitive tests.In study 1, 48 older adults completed two touchscreen cognitive tests, on three occasions, in controlled conditions, alongside a battery of standard tests of cognitive functions. In study 2, 40 older adults completed the same two computerized tasks on multiple occasions, over three separate week-long periods, in their own homes, without a researcher present. Here, the tasks were incorporated into a wider touchscreen system (Novel Assessment of Nutrition and Ageing (NANA)) developed to assess multiple domains of health and behavior. Standard tests of cognitive function were also administered prior to participants using the NANA system.Performance on the two “NANA” cognitive tasks showed convergent validity with, and similar levels of reliability to, the standard cognitive battery in both studies. Completion and accuracy rates were also very high. These results show that reliable and valid cognitive data can be collected from older adults using unsupervised computerized tests, thus affording new opportunities for the investigation of cognitive.

## Introduction

Age-related changes in cognitive function can be examined over various timescales. Most commonly, change is considered over relatively long periods, such as months or years, for instance when monitoring the rate of decline associated with neurodegenerative conditions (Patterson et al. 1999) or to examine improvements in function following an intervention (Antunes et al. 2015). At the other end of the continuum, moment-to-moment variability in performance (i.e., over seconds or minutes) can be assessed using indices such as the standard deviation of reaction times in speeded response time tasks (Jensen 1992). Such moment-to-moment variability is known to increase with age (Li et al. 2004) and has been associated with diverse health and functional outcomes, including increased risk of falling (Graveson et al. 2015), everyday behavioral mistakes (Steinborn et al. 2015), as well as future mortality (Shipley et al. 2007) and cognitive decline (MacDonald et al. 2003).

Variability in cognitive function can also be considered at intermediate or “micro-longitudinal” (Palmier-Claus et al. 2011) timescales, such as over hours or days. For instance, diurnal variability in cognitive processing is a robust phenomenon in all age groups (Baddeley et al. 1970). Importantly, the extent of this variability is known to increase with age (May et al. 1993) and in people with cognitive impairment (Paradee et al. 2005), indicating its relationship with health status. Fluctuations in cognitive performance over periods of hours or days are also characteristic of some acute health conditions, such as delirium (American Psychiatric Association 2013), and with physiological changes, such as altered levels of ammonia (Balata et al. 2003) and glucose (Somerfield et al. 2004) in the blood, demonstrating the importance of micro-longitudinal changes as general health indicators. Furthermore, the physiological mechanisms underlying micro-longitudinal patterns are believed to differ from those underpinning shorter-term, moment-to-moment variability (Schmiedek et al. 2013), and so may offer unique information about the mechanisms underlying age-associated changes in cognition and health (Gamaldo and Allaire 2015).

Compared with research over macro (i.e., months-years) or moment-to-moment timescales, knowledge of the nature and relevance of micro-longitudinal patterns of function are relatively limited (Gamaldo and Allaire 2015; Schmiedek et al. 2013). One reason for this is the practical difficulties associated with performing repeated cognitive assessments. First, there are the general issues associated with all repeated cognitive testing, such as accounting for practice effects (Bird et al. 2004) and producing multiple sets of equivalent stimuli (Sullivan 2005). However, more challenging is the high density of assessments needed to track patterns of performance over multiple sessions, which can result in high levels of burden and inconvenience to participants, and cost to the researcher or clinician. These issues are further multiplied in studies that involve the monitoring of additional health or behavioral variables, such as when investigating their temporal associations with cognitive change. To advance our understanding of micro-longitudinal patterns of function, there is therefore a need for assessment methods that enable repeated measures of cognition to be taken over periods of hours and days, and that place low burden on participants, researchers, and clinicians.

In order to address this, we developed the Novel Assessment of Nutrition and Ageing (NANA) toolkit, which is touchscreen-based software for tracking cognitive function, as well as other health and behavioral domains, across micro-longitudinal timescales (Astell et al. 2014). To minimize the cost and burden of micro-longitudinal assessment, the NANA system was specifically developed with older participants, for them to use in their own homes, without a researcher being present. The cognitive tasks were designed to be particularly sensitive to cognitive processing speed, which is known to be indicative of a broad range of health and well-being outcomes in later life (Lara et al. 2013). Age-related declines in processing speed have also been shown to account for a large proportion of variance in other cognitive tasks (Tucker-Drob 2011), and thus provide an efficient way of gathering informative indicators of cognitive change.

Self-administered computerized tests have already shown promise as a feasible way of collecting valid, single assessments of cognitive function in older adults in experimental situations (Tierney et al. 2014). However, the performance of such tests has not yet been examined over micro-longitudinal time periods, in unsupervised settings. In this paper, we therefore address the question of whether it is possible to collect reliable and valid cognitive data over micro-longitudinal timescales, without a researcher being present. We do this by assessing the performance of two NANA cognitive tasks under both controlled and naturalistic conditions. In study 1, we assessed the usability, validity, and reliability of the NANA cognitive tasks when administered in a supervised, laboratory-based environment, but with minimal researcher involvement. In study 2, we assessed the performance of these tasks when used by older adults, unsupervised, in their own homes to collect data over micro-longitudinal timescales. The validity of the tasks as measures of age and health-relevant cognitive function was assessed by examining the extent to which performance on the NANA cognitive tasks correlated with performance on standard tests of cognitive processing speed, as well as tests of higher cognitive functions (episodic memory and executive function), and participant age. Reliability was determined by examining correlations and changes in performance over time.

## Study 1

### Methods

#### Participants

Forty-eight community-living adults (17 males) aged 65–89 years (mean = 72 years) provided written informed consent to participate in this study, which had been approved by the Fife and Forth Valley Committee on Medical Research Ethics (Ref: 08/S0501/104) and the University of St Andrews Teaching and Research Ethics Committee.

### NANA cognitive function tasks

Two touchscreen tasks (the Shopping List task and the Squares task) were programmed in Embarcadero Delphi 2010 and administered on a 15″ touchscreen computer (Asus EeeTop, model ET1610PT). The Shopping List task was designed to draw on a broad range of cognitive functions that are known to be markers of age and health. In particular, the task was modeled on principles of symbol substitution tasks that require participants to use a digit-symbol pairing key to identify the corresponding symbols for a series of stimuli as quickly as they can (Lezak et al. 2012). Performance on these tasks is believed to depend on a range of cognitive functions, including attention (Strauss et al. 2006) and processing speed (Deary et al 2010).

At the start of the Shopping List task, the instruction to “Report what is on the shopping list as quickly as you can” was presented on the screen. The instruction remained on the screen until the participant touched a box containing the word “start.” Following this, a screen containing a “shopping list” in the top right quadrant of the screen, and four response boxes (containing the numbers 2–4) along the bottom of the screen, was presented. The shopping list was a white box containing the names of four items (apples, carrots, lemons, onions), each preceded by one of the numbers 2, 3, 4, or 5. The order of the four items, and of the numbers preceding them, was randomly determined each time the task was administered, and then remained the same for the duration of the task.

**Fig. 1:**
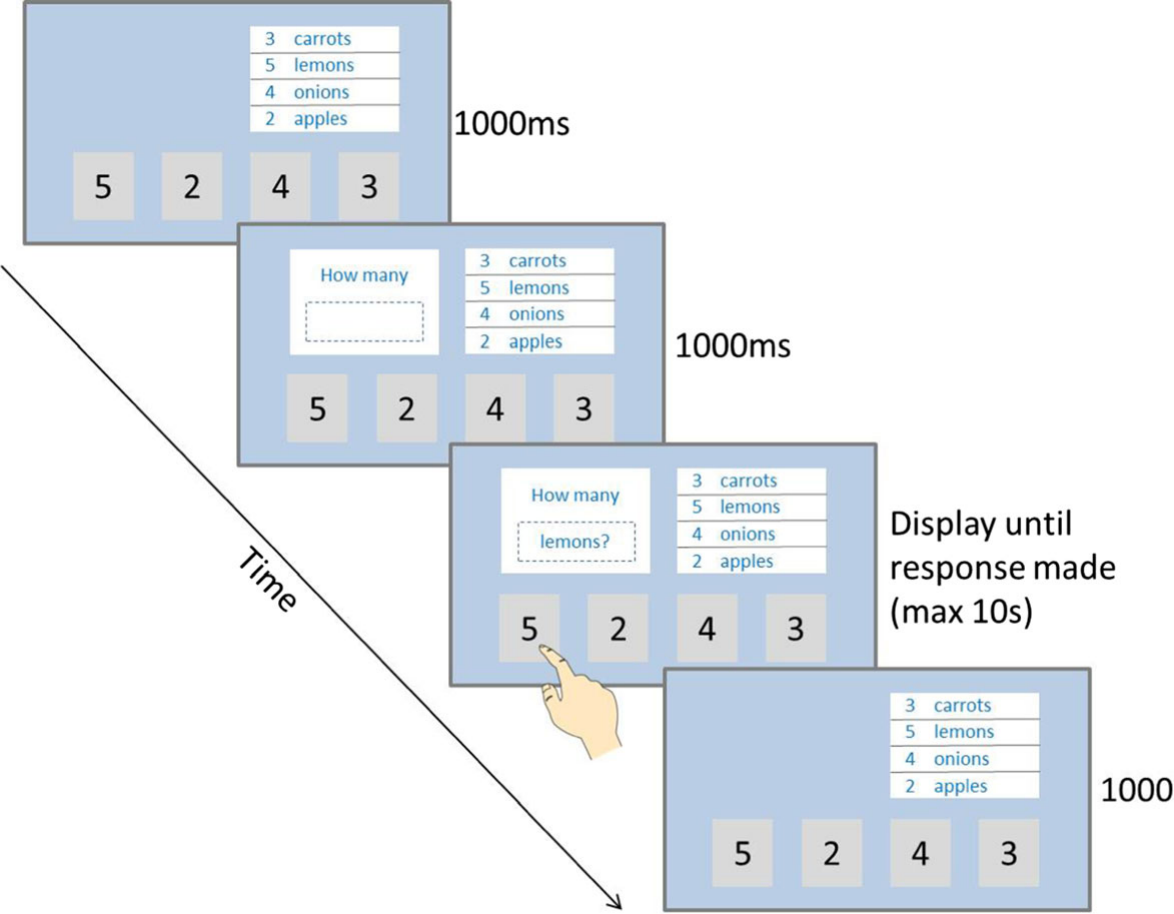
Schematic diagram of a trial in the “random” version of the Shopping List task

After a 1000-ms delay, the first trial was presented. For this, a white box containing the question stem “How many” with an empty box below it was first presented in the top left quadrant of the screen for 1000 ms. The name of one of the items on the shopping list (appended with a question mark) was then presented in the box underneath the question stem. This display remained on screen until the participant touched one of the response boxes at the bottom of the screen (or for a maximum duration of 10 s if no response was made). Following a response, the question text and surrounding box were removed from the screen for 1000 ms, and then, the second trial began. The shopping list and response boxes remained on screen for the duration of the task. An example of a Shopping List task trial is shown in Fig. 1.

As there have been no previous published examples of symbol substitution tasks that require touchscreen responses, we created two different response option formats so that we could determine which format led to the best psychometric test properties. In one version of the task, the response options were presented in an ascending order (i.e., 2, 3, 4, 5), and in the other version, they were presented in a random order. For the random order version, a new random order was created each time the task was administered, and the same random order was then retained for the duration of the task. Each participant completed 20 trials of each version of the task. The order in which participants completed these two versions was counterbalanced between participants so that psychometric properties of each version could be compared with one another. In each 20-trial iteration of the task, each of the four items on the shopping list (apples, carrots, lemons, onions) was presented five times. The order in which the items were presented was randomly determined, with the restriction that the same item was never presented twice in succession. This was to minimize confusion to participants from being asked the same question twice in a row.

Performance on the Shopping List task was assessed according to the accuracy of responses (i.e., the proportion of correct responses made) and average response time of correct responses in each session. Median rather than mean response times were calculated for each participant in order to minimize the effects of extreme values (Jensen 1992).

The Squares task was a speeded choice response time task, a measure of cognitive processing speed (Deary et al. 2010). This task was designed to be simpler to understand than the Shopping List task in case participants struggled to complete the Shopping List task in unsupervised settings.

**Fig. 2:**
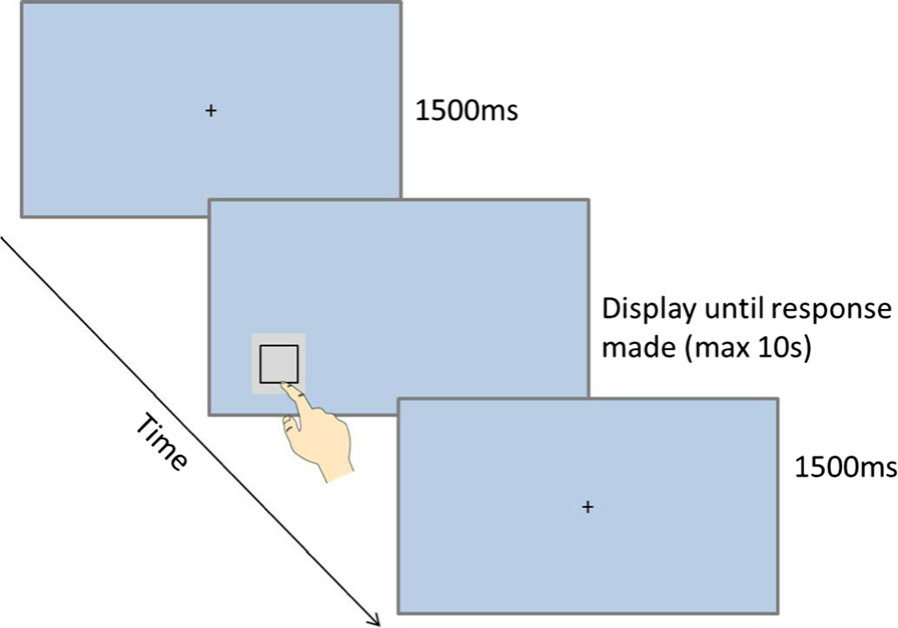
Schematic diagram of a trial in the Squares task

At the start of the Squares task, the instruction “Touch the boxes as quickly as you can” was presented on the screen. The instruction remained on screen until the participant touched a box containing the word start. The first trial then began. For this, a black fixation cross was presented in the center of the screen for 1500 ms. The fixation cross then disappeared, and a gray box (containing a black square) was presented in one of the four locations along the bottom of the screen. The four possible locations and the sizes of the response boxes were the same as those presented in the Shopping List task. The response box disappeared after it had been touched (or after a maximum duration of 10 s, if no response was made). The next trial then began with the fixation cross again being presented. Figure 2 shows a schematic example of a trial in the Squares task.

Each participant performed 20 trials of the Squares task. In each 20-trial session, each of the four locations was presented five times. The order in which the response boxes were presented was randomly determined, with the restriction that the same location was not presented more than three times in succession. Performance was assessed by calculating median response times for each session.

### Battery of standard measures

A battery of standardized cognitive tests was also administered so that the concurrent validity of the NANA tasks could be established. Four of these tests provided measures of processing speed, which is considered fundamental to many other higher-order cognitive functions (Tucker-Drob 2011) and to be sensitive to age-related change (Lara et al. 2013). These were as follows:

1. A computerized Speeded Reaction Time task adapted from the PEBL battery (Mueller 2009), in which participants were asked to make a speeded keyboard key press each time they saw a black cross in the middle of the computer screen. After two short practice blocks, participants performed two blocks of 15 trials each. The time between a response being made and the next stimulus being presented varied from 1400 to 3200 ms. Performance was measured as the median response time of responses made within the valid time window of 150–3000 ms across the two blocks.
2. The Symbol Digit Modalities Test (SDMT: Smith 1982), in which participants were required to write the corresponding number for each of a series of abstract symbols, according to a number-symbol key printed at the top of the page. The number of correct responses made in a 90-s period was recorded.
3. A Number Copy Task, in which participants were asked to simply copy randomly generated sequences of the digits 1–9. This task was scored according to the number of correct responses made in 30 s.
4. Part A of the Trail Making Test (Spreen and Strauss 1998), in which participants are asked to join together numbered circles as quickly as they can. This task was scored as the time taken to correctly join all 25 circles, with any mistakes being called to the participant’s attention by the researcher during task performance.

Executive functions were assessed using three measures.

1. Part B of the Trail Making Test (Spreen and Strauss 1998) was used to assess the task-switching component of executive function. In this task, participants are asked to join together a series of numbered and lettered circles, alternating between numbers and letters. This task is scored in the same way as part A, with shorter completion times indicating better performance.
2. A forwards and backwards digit span task (Lezak et al. 2012) was used as a measure of working memory. For this, the length of the string started at two digits, and then increased by one digit every two trials to a maximum length of nine digits for the forward span task, and eight for the backward span task. The task was discontinued if the participant failed both items of a given string length. The number of correct responses made to the forwards and backwards task was summed together to give a total digit span score.
3. A Stroop task (Stroop 1935) was administered to assess inhibitory executive functions. This task was administered in three parts: first participants were given a sheet containing 16 rows of 6 rectangles, each colored red, blue, or green, and were asked to name the color that each rectangle was printed in as quickly as they could. For the second part, the rectangles were replaced with the neutral words “when,” “and,” and “hard,” and participants had to name the color that the words were printed in. In the third part, the neutral words were replaced with the color words “red,” “blue,” and “green,” which were always incongruent with the color that the words were printed in. The number of correct responses made in 30 s was recorded for each part. A measure of interference was then calculated for each participant by dividing the number of correct responses made in the third part by the number made in the second part. Lower interference scores therefore indicate a higher amount of interference.

Verbal episodic memory was assessed using a word recall task. For this, 15 words from the Rey Auditory-Verbal Learning Test (Lezak et al. 2012) were read aloud three times, and the participant was asked to recall as many words as possible each time. A score for immediate recall was calculated by summing the number of words correctly recalled on each of the three occasions. After a delay of approximately 20 min, the participant was again asked to recall as many of the words as possible. The number of words correctly recalled on this occasion was recorded as the delayed recall score.

Two additional tests were included as measures of global cognitive function and prior cognitive ability, respectively. The Mini Mental Status Examination (MMSE: Folstein et al. 1975) contains a series of brief tasks designed to screen for cognitive impairment. It is scored out of 30, with scores below 24 generally taken as an indicator of potential impairment (Iverson 1999). The National Adult Reading Test (NART: Nelson, 1982) requires participants to read aloud a series of 50 words with irregular pronunciations, providing a proxy measure of reading ability that is indicative of prior intellectual functioning (Crawford et al. 2001). The task is scored according to the number of errors made, and performance has been shown to be relatively resistant to dementia (McGurn et al. 2004) and short-term cognitive disturbance (Brown et al. 2011).

### Procedure

Each participant was invited to attend three separate, individual testing sessions, over a week-long period, so that the validity and reliability of the tasks over micro-longitudinal timescales could be established. In the first session, participants provided demographic details, as well as details about their current use of computers, and completed the short-form Geriatric Depression Scale (Sheik and Yesavage 1986). Participants then completed the NANA tasks and standard measures of cognitive function. The order in which participants completed the NANA and standard measures was counterbalanced between participants in order to allow performance on the two sets of tasks to be fairly compared with one another.

Prior to starting the NANA tasks, participants completed a brief process of familiarization with the touchscreen by undergoing a series of practice operations that involved making touchscreen responses to on-screen instructions. When completing the NANA tasks, participants were asked to follow the simple instructions on screen and make their responses by touching the appropriate part of the screen. As the tasks were being developed for future unsupervised use, the researcher who administered the tasks minimized additional contact with the participant while they completed the tasks, and only provided additional clarification or reassurance when absolutely necessary.

In addition to the NANA tasks described above, each participant also completed a number of other short touchscreen measures of cognition, mood, and appetite that were also being considered for inclusion in the NANA system during the session. The additional cognitive tasks were not selected for further development, and so are not reported here. The validation of the mood and appetite measures is reported elsewhere (Brown et al. 2016).

The NANA tasks and a subset of the standard measures that were suitable for repeated testing (see Table 3) were repeated on each of the subsequent two testing sessions. All participants received a commemorative study mug at the end of their first session, as well as a £5 (approximately $7.5) expense payment for each session they completed.

### Data analysis

As a number of cognitive tasks produced data that were ordinal, not normally distributed, and/or had outliers, non-parametric tests of correlation and difference were used for all analyses. Kendall’s Tau tests were used rather than Spearman’s rank to assess correlations as the former are better suited to data containing several tied ranks (Field 2013), which was the case with a number of the variables. Correlation values produced by Kendall’s Tau tests tend to be lower than those of Spearman’s rank due to the different way in which they are calculated (Capéraà and Genest 1993).

In order to assess the concurrent validity of the NANA tests, the degree of correlation between participants’ performance on each of the NANA tasks and the standard cognitive battery in the first testing session was calculated. In order to assess test-retest reliability of the NANA tasks, the degree of correlation between performance across sessions was calculated. As we were expecting cognitive function to vary over micro-longitudinal timescales, it is not possible to assess reliability from these values alone. Therefore, for comparability, between-session correlations were also calculated for each of the standard cognitive function tasks that were administered on each testing session. Friedman tests of difference were also performed for each task to determine whether any change in performance occurred over the three testing session, for instance due to practice effects.

## Results

### Participant characteristics

A procedural error meant that two participants used a different model of computer to perform the NANA tasks, and so, their data were excluded from these analyses. The remaining 46 participants (16 males) had a mean age of 72 years (SD = 5.9). Their mean MMSE score was 28.1 (SD = 1.98), and their mean GDS score was 1.32 (SD = 1.73), indicating low levels of cognitive impairment and depression. Two of these participants (both female) were not able to attend a third testing session within the time period of the study, and so only contributed data to the first and second testing sessions.

Education levels among participants were generally high: 46 % were educated to degree level or above, a further 22 % held professional or semi-professional qualifications, 15 % were educated up to the equivalent of A-level, 11 % up to the equivalent of GCSE, and just 7 % held no educational qualifications. Self-reported levels of computer use were also high, with the majority of participants (74 %) reporting using them on most days, and a further 15 % using them up to 5 days per week. In response to a question asking how competent they felt when using computers without assistance, five participants (11 %) selected the option “very,” 23 (50 %) selected “fairly,” 11 (24 %) selected “a little,” and just seven (15 %) selected “not at all.”

### NANA task performance

Only two participants failed to respond to a single trial of the Shopping List task (one during the second testing session and one during the third), and no participant failed to respond to any trials in the Squares task. The accuracy of participants’ responses was very high in both versions of the Shopping List task and did not differ across testing sessions (Table 1).

**Table 1:** Accuracy rates for the ascending and random order versions of the Shopping List task in each testing session of study 1

	Session 1 % accuracy of responses	Session 2 % accuracy of responses	Session 3 % accuracy of responses	Friedman χ^2^ (d.f. = 2)
	Mean (range)	Mean (range)	Mean (range)	
Ascending order	98.41 (85–100)	98.86 (80–100)	98.52 (85–100)	1.25, *p* = 0.53
Random order	98.18 (80–100)	98.30 (90–100)	98.64 (90–100)	0.79, *p* = 0.67

The results of the correlation analyses between performance on all of the NANA and standard cognitive tasks and participant age are shown in Table 2. They show that performance on each of the NANA tasks correlated significantly with almost all of the standard tasks of cognitive function. Of the NANA tasks, the random version of the Shopping List task showed the strongest pattern of correlation with the standard cognitive function tasks. As expected, performance on this task was particularly well correlated with performance on the Symbol Digit task, indicating high levels of similarity in the cognitive operations involved. Correlations between the NANA tasks and the NART measure of prior cognitive ability were generally lower than with the measures of current cognitive function, indicating that the NANA tasks were better measures of current, rather than prior, cognitive ability. As with most of the standard cognitive tasks, all of the NANA tasks also correlated with age, showing their sensitivity to age-related change.

**Table 2:** Kendall’s Tau correlation coefficients between session 1 performance on the NANA tasks, standard cognitive battery, and participant age in study 1

		1	2	3	4	5	6	7	8	9	10	11	12	13	14
1	SL Asc	-	-	-	-	-	-	-	-	-	-	-	-	-	-
2	SL Ran	.53^***^	-	-	-	-	-	-	-	-	-	-	-	-	-
3	SqT	.32^**^	.47^***^	-	-	-	-	-	-	-	-	-	-	-	-
4	MMSE	-.30^**^	-.41^***^	-.36^**^	-	-	-	-	-	-	-	-	-	-	-
5	SDMT	-.55^***^	-.61^***^	-.40^***^	.43^***^	-	-	-	-	-	-	-	-	-	-
6	NC	-.28^**^	-.30^**^	−.24^*^	.26^*^	.42^***^	-	-	-	-	-	-	-	-	-
7	Im Rec	-.37^***^	-.38^***^	-.25^**^	.29^**^	.45^***^	.30^**^	-	-	-	-	-	-	-	-
8	Del Rec	-.36^***^	-.41^***^	-.30^**^	.42^***^	.52^***^	.29^**^	.69^***^	-	-	-	-	-	-	-
9	DS	-.38^***^	-.44^***^	−.20^*^	.32^**^	.44^***^	.27^**^	.28^**^	.35^**^	-	-	-	-	-	-
10	Trails A	.43^***^	.35^***^	.27^**^	-.28^**^	-.36^***^	-.30^**^	-.26^**^	-.31^**^	-.48^***^	-	-	-	-	-
11	Trails B	.42^***^	.42^***^	.26^**^	-.27^**^	-.51^***^	-.32^**^	-.34^***^	-.32^**^	-.40^***^	.37^***^	-	-	-	-
12	Stroop	-.29^**^	-.28^**^	−.23^*^	.24^*^	.26^**^	.11	.15	.24^*^	.22^*^	-.25^**^	-.17	-	-	-
13	SRT	.20^*^	.24^*^	.34^**^	−.22^*^	-.26^**^	−.10	−.09	−.18^*^	−.19^*^	.21^*^	.25^**^	-.18^*^	-	-
14	NART	.15	.25^**^	.20^*^	-.28^**^	-.27^**^	−.15	−.24^*^	−.25^**^	−.19^*^	.19^*^	.31^**^	−.09	.25^*^	-
15	Age	.29^**^	.36^***^	.37^***^	-.31^**^	-.33^**^	-.50^***^	-.28^**^	-.25^*^	-.20^*^	.24^*^	.23^*^	-.24^*^	.07	.03

*SL Asc* Shopping List task with ascending response order, *SL Ran* Shopping List task with random response order, *SqT* Squares Task, *MMSE* Mini-Mental Status Examination, *SDMT* Symbol Digit Modality Test, *NC* Number Copy, *Im Rec* Immediate Recall, *Del Rec* Delayed Recall, *DS* Digit Span, *SRT* Speeded Reaction Time, *NART* National Adult Reading Test, *GDS* Geriatric Depression Scale, *PA* Positive Affect, *NA* Negative Affect. ^*^ *p* < 0.05; ^**^ *p* < 0.01; ^***^ *p* < 0.001 (for one-tailed tests)

The results of the reliability analyses are shown in Table 3. They show that cross-session correlations in performance were significant for all of the NANA and standard cognitive tasks. The strength of the correlation coefficients for the NANA tasks were similar in magnitude to those of the speeded reaction time task, indicating comparable levels of reliability.

**Table 3:** Mean performance levels for the NANA and standard cognitive tasks that were administered in all three testing sessions in study 1

Task		Session 1 Mean (SD)	Session 2 Mean (SD)	Session 3 Mean (SD)	Friedman χ^2^ ^b^	Cross-session correlations (Kendall’s Tau values)		
	*N* ^a^					1 vs 2	2 vs 3	1 vs 3
SL Asc (ms)	44	1688 (334)	1654 (347)	1574 (270)	13.84, *p =* .001	.56, *p <* .001	.57, *p <* .001	.63, *p <* .001
SL Ran (ms)	44	1797 (330)	1782 (343)	1736 (296)	3.90, *p =* .14	.60, *p <* .001	.59, *p <* .001	.62, *p <* .001
SqT (ms)	43	518 (78.9)	522 (80.1)	496 (68.5)	9.73, *p =* .007	.68, *p <* .001	.65, *p <* .001	.61, *p <* .001
SDMT (no. correct)	44	44.18 (10.31)	47.59 (12.41)	50.61 (13.44)	30.85, *p <* .001	.77, *p <* .001	.78, *p <* .001	.77, *p <* .001
NC (no. correct)	44	49.48 (10.13)	50.20 (10.82)	50.18 (11.52)	2.24, *p =* .33	.77, *p <* .001	.82, *p <* .001	.75, *p <* .001
DS (no. correct)	44	18.57 (4.57)	18.80 (4.51)	19.18 (5.04)	1.11, *p =* .58	.66, *p <* .001	.72, *p <* .001	.61, *p <* .001
STROOP interference	44	0.64 (0.11)	0.68 (0.14)	0.63 (0.14)	7.28, *p =* .03	.29, *p =* .003	.25, *p =* .01	.18, *p =* .047
SRT (ms)	42	317 (55.4)	327 (108.1)	324 (72.6)	1.10, *p =* .59	.63, *p <* .001	.63, *p <* .001	.63, *p <* .001

*SL Asc* Shopping List Task with ascending response order, *SL Ran* Shopping List Task with random response order, *SqT* Squares Task, *SDMT* Symbol Digit Modality Test, *NC* Number Copy, *DS* Digit Span, *SRT* Speeded Reaction Time

^a^Note that 44 participants completed all three testing sessions. However, the SqT was not administered to one participant, and the SRT was not administered to two participants

^b^Exact test used to calculate significance

Some of the NANA tasks and standard cognitive tasks also showed evidence of significant improvements in performance across the sessions. Pairwise Wilcoxon signed rank tests (one-tailed, uncorrected for multiple comparisons) showed that, for the ascending version of the Shopping List task and the Squares task, there were significant improvements in performance between sessions 2 and 3 (*Z* = 3.18, *p* = 0.001; *Z* = 3.20, *p* < 0.001, respectively), but not between sessions 1 and 2 (*Z* = 1.21, *p* = 0.12; *Z* = 0.14, *p* = 0.45, respectively). For the Stroop task, significant reductions in interference were seen between sessions 1 and 2 (*Z* = 2.03, *p* = 0.02), but not between sessions 2 and 3 (*Z* = 1.43, *p* = 0.08). For the Symbol Digit task, significant improvements were seen between sessions 1 and 2 (*Z* = 3.67, *p* < 0.001) and between sessions 2 and 3 (*Z* = 3.25, *p* < 0.001). There were no significant changes in performance across sessions for the random version of the Shopping List task (Table 3).

## Discussion

Both the Shopping List and the Squares task show validity as reliable tests of processing speed, which has been considered a biomarker of cognitive aging (Deary et al. 2010). There were also significant associations with measure of executive function and verbal episodic memory, perhaps reflecting the fundamental role of processing speed in these higher cognitive abilities (Tucker-Drob 2011), as well as with participant age. Although there was evidence of practice effects for the ascending version of the Shopping List task and the Squares task, no significant practice effects were observed for the random version of The Shopping List task. This task also showed larger correlations with the standard cognitive tasks and participant age than the other NANA tasks, making it the psychometrically strongest of the three.

## Study 2

The aim of study 2 was to examine the performance of the two NANA cognitive tasks when used by participants, in their own homes, without a researcher being present, over micro-longitudinal timescales. This was done as part of a larger validation study of the entire NANA toolkit, which included computer-based measures of participants’ dietary intake, mood, appetite, grip strength, physical activity, and exhaustion (Astell et al. 2014).

## Methods

### Participants

Forty community-living adults (24 female, 16 male) aged 64–88 years (mean age = 72 years) gave written informed consent to participate in this study, which had been approved by the Fife and Forth Valley Committee on Medical Research Ethics (Ref: 08/S0501/104) and the University of St Andrews Teaching and Research Ethics Committee.

### NANA cognitive function tasks

The two NANA cognitive tasks were again administered on a 15″ touchscreen Asus EeeTop computer (model ET1610PT), which formed part of the NANA system hardware. In addition to the cognitive tasks, the NANA system was being used to record dietary intake, physical activity, mood, appetite, grip strength, and exhaustion (Astell et al. 2014). A webcam (used for photographing participants’ dietary intake as part of the dietary assessment function of NANA) was therefore attached to the top of the computer. In order to integrate the cognitive tasks into the NANA software, they were re-programmed (in C#). During this integration period, some changes to the software were made, as detailed below.

As the psychometric properties of the random version of The Shopping List task were shown to be better than those of the ascending version, all of the response options for this task were presented in a randomly determined order in study 2. As in study 1, a random order was created each time the task was administered, and this same random order was then retained for the duration of the task. The response options were presented along with the shopping list, in white text in light gray boxes. The light gray color was used to indicate that the response buttons were not yet active. After the “how many” question stem had been presented for 2000 ms, one of the four shopping list items was presented, and the color of the response boxes turned from gray to green, to indicate that they were now active. The text remained on screen until a response was made or for a maximum of 15 s if no response made. This “timeout” period was 5 s longer than in study 1 to allow for a greater range of response times that might occur in the unsupervised test setting. After the question stem and word disappeared from the screen, the color of the response buttons turned back to gray for 1000 ms, before the next trial began. Ten, rather than 20, trials were presented in each iteration of the task due to the larger number of tasks that participants were required to complete in this study. The identity of the food item was randomly determined on each trial, with the restriction that the same food name was never presented twice in succession.

For the Squares task, the initial instruction was altered to “Touch the white squares as quickly as you can” to reflect the different color of the stimuli. As with the Shopping List task, the locations of the four response buttons were presented on screen throughout the task. They remained gray for the first 1500 ms of each trial, and then turned from gray to green to indicate that they were active. When they turned green, a white square was presented in one of the four boxes. The boxes remained green until the participant made a response, or for a maximum of 15 s if no response was made. All four boxes then reverted to the light gray color (with no white square) for 1500 ms, before the next trial began. Four rather than one response option were presented so that accuracy as well as speed of responses could be measured. As the response boxes remained on screen throughout this task, no fixation crosses were presented between trials. Ten trials were presented in each iteration of the task. The location of the white square was randomly determined on each trial, with the constraint that no two consecutive trials were the same.

### Standard cognitive battery

A subset of the standardized cognitive tests used in study 1 was also administered to participants to assess concurrent validity of the NANA tasks. This battery comprised the following: the SDMT (Smith 1982), Number Copy Task and Part A of the Trail Making Test (Spreen and Strauss 1998) to measure processing speed; Part B of the Trail Making Test (Spreen and Strauss 1998) to measure executive function; the immediate and delayed recall parts of the Word Recall task to measure verbal episodic memory; the MMSE (Folstein et al. 1975) to measure global cognitive function; and the NART (Nelson 1982) to measure prior cognitive ability.

### Procedure

Each participant was given the NANA system to use in their home for three periods, each of approximately 7 days in duration, and with a break of approximately 3 weeks between each period of use. At the start of the first period of use, the participant was given the chance to practice brief versions of all the tasks and assessments in the presence of a researcher until they felt comfortable using the system. They were also given a simple manual for the system and a researcher’s contact number to use if they had problems.

In each period of use, participants were asked to use the NANA system to record everything they ate and drank, as well as to perform various assessments of their physical activity, grip strength, exhaustion, mood, appetite, and cognitive function. The two NANA cognitive tasks were scheduled to be administered once per day, following some brief assessments of self-reported mood and appetite (Brown et al. 2016). Participants were prompted to perform these tasks when they interacted with the system by an on-screen message indicating that readings or exercises were due. They were given the option to complete the tasks then or postpone them to later. When an assessment was not completed before the next one was due, multiple assessments would be administered within a single session, in the same order that they had been scheduled to be completed. Postponed assessments continued to be shown on the system until completed. The number of cognitive assessment due was denoted by a digit on an icon of a head and cogwheel silhouette in the bottom left hand corner of the screen.

The standard cognitive test battery was administered at baseline (i.e., before the NANA system was installed in the participant’s home) and again at the end of the third period of use. Other data (including measures of depression, weight, physical functioning, and blood and urine analysis) were also collected at times before and after the periods of NANA system use, as part of the wider system validation. These are reported in detail in Astell et al. (2014) and Timon et al. (2015).

### Data analysis

As with study 1, some of the datasets did not meet the assumptions for parametric analysis, and so, non-parametric tests of difference and correlation were used for all analyses. In order to determine whether participants were able to understand and complete the tasks, average response rates and accuracy levels on each of the NANA tasks were calculated for each participant in each of the three testing periods. In order to determine the validity of the NANA tasks, Kendall’s Tau tests were used to establish the degree of correlation between performance on the NANA tasks with scores on the standard cognitive battery and participant age.

As we were interested in establishing the validity of the tests during a single session as well as across a longer period of time, two different time periods of data collection were assessed: first, each participant’s average response time across the whole of the first testing period was calculated. This was done by first calculating their median response time of the 10 trials administered for each test session, and then taking the mean of these values from all of the sessions completed during the first weekly session. The first testing period was selected due to having the closest temporal proximity with the baseline pen and paper cognitive tasks used to assess validity. Second, in order to examine validity for a single test session, the median response time for a single test session from the middle of the first testing period was extracted. For both of these calculations, only response times to correct trials were included.

In order to determine the reliability of the NANA tasks, Kendall’s Tau tests were used to examine the degree of correlation between average response times across the three testing sessions. Friedman and Wilcoxon tests of difference were used to determine whether any changes in reaction time occurred across the three testing sessions.

## Results

### Participant characteristics and data collection

The mean MMSE score of the participants was 28.63 (SD = 1.64). Thirty-six of the participants (90 %) reported that they had previously used a computer, and 32 (80 %) reported being Internet users.

Although each participant was scheduled to complete seven sessions of each NANA cognitive task in each testing period, in some cases, the number of datasets collected was greater or less than this. Reasons for this included the following: administrative errors that resulted in the systems being collected too early, or the wrong number of trials being programmed; technical problems with the systems that led to additional trials being presented; and participants being away from home for part of the measurement period. Some of the datasets that were collected for each task were also subsequently excluded from the final analyses. Specifically, 42 datasets for each of the tasks (5.09 % of total datasets) were excluded as they were collected within 15 min of a previous data collection period, and therefore not considered to truly represent a separate period of assessment. An additional four data sets for the Shopping List task and one for the Squares task were also excluded as data from the corresponding cognition or mood tasks had not been collected, indicating an anomaly with the data collection session. The final analysis therefore related to 781 datasets (each containing one participant’s responses to 10 trials of the Shopping List task and 10 trials of the Squares task): 248, 264, and 269 datasets from the first, second, and third testing period, respectively.

### NANA task performance

As can be seen in Table 4, response rates were very high for both tasks, with far less than 1 % of trials “timing out” before a response was made. Average accuracy rates were also very high and showed no significant changes across the three testing periods (Table 4). Only two participants performed one of the tasks at accuracy levels below 40 % (one completed the Shopping List task on one occasion with an accuracy rate of 10 %, and another completed the Squares task on one occasion with an accuracy rate of 30 %, and these were both in the first task session of the first testing period), and these data were removed from the subsequent validity and reliability analyses. All other tasks were completed at accuracy rates above this, demonstrating that participants were able to understand and complete the tasks, even without a researcher being present.

**Table 4:** Mean percentages of correct, incorrect, and “timed out” responses made for each NANA task in each of the three testing periods of study 2

		Period 1	Period 2	Period 3	Friedman χ^2^ (d.f. = 2)
		Mean (*range*)	Mean (*range*)	Mean (*range*)	
Shopping List Task					
	Mean % of correct responses	98.36 (71–100)	98.13 (86–100)	98.37 (90–100)	1.96, *p* = .38
	Mean % of incorrect responses	1.42 (0–22)	1.23 (0–7)	1.39 (0–10)	0.56, *p* = .77
	Mean % of timed out trials	0.22 (0–7)	0.64 (0–13)	0.25 (0–2)	2.48, *p* = .33
Squares Task					
	Mean % of correct responses	99.55 (89–100)	99.89 (97–100)	99.51 (96–100)	5.69, *p* = .056
	Mean % of incorrect responses	0.42 (0–11)	0.08 (0–2)	0.34 (0–3)	4.00, *p* = .13
	Mean % of timed out trials	0.04 (0–1)	0.04 (0–1)	0.15 (0–4)	0.67, *p* > .99

As can be seen in Table 5, response times in the Shopping List and Squares tasks during testing period 1 were significantly correlated with performance on all of the baseline cognitive tests and participant age and at similar levels to those seen in study 1. As in study 1, the strongest correlations were with the SDMT (processing speed) task, and the strength of correlations was greater for the Shopping List than the Squares task. The correlations for response times in single task sessions were also significant in most cases, although were generally of a lower magnitude than for performance averaged across the testing period. However, the single session response time data for the two NANA tasks were significantly and strongly (*r*_τ_ = .49, *p* < 0.001) correlated with one other. Taken together, these results are consistent with performance averaged across the session providing a valid measure of average processing speed, and performance within a single session providing a valid measure of “momentary” processing speed.

**Table 5:** Kendall’s Tau correlation coefficients between performance on the NANA tasks and each of the baseline standard cognitive tasks and participant age for study 2

	SL Single session (*N* = 39)	SqT Testing period1 (*N* = 39)	SqT Single session (*N* = 39)	MMSE (*N* = 39)	SDMT (*N* = 39)	NC (*N* = 39)	Im Rec (*N* = 39)	Del Rec (*N* = 39)	Trails A (*N* = 38)	Trails B (*N* = 35)	NART (*N* = 39)	Age (*N* = 39)
Shopping List task												
Testing period 1 (mean of daily median RTs)	.63^***^	.64^***^	.49^***^	−.29^*^	−.60^***^	−.42^***^	−.31^**^	−.35^**^	.43^***^	.44^***^	.33^**^	.36^**^
Single session (median of middle session RTs)	-	.49^***^	.49^***^	−.16	−.41^***^	−.33^**^	−.23^*^	−.23^*^	.21^*^	.15	.32^**^	.31^**^
Squares Task												
Testing period 1 (mean of daily median RTs)	.49^***^	-	.64^***^	-.21^*^	-.44^***^	-.37^**^	-.20^*^	-.23^*^	.38^***^	.43^***^	.25^*^	.29^**^
Single session (median of middle session RTs)	.49^***^	.64^***^	-	-.12	-.35^**^	-.23^*^	-.18	-.11	.21^*^	.25^*^	.15	.27^*^

Data for the NANA tasks are shown separately for the average performance across testing period 1 and for a single session from the middle of testing period 1

^*^ *p* < .05; ^**^ *p* < .01, ^***^ *p* < .001

Table 6 shows the results of the reliability analyses for the 38 participants who contributed data to all three testing sessions. As with study 1, cross-session correlations in performance were significant for all tasks. The strengths of the correlations were generally greater than in study 1, probably because the average session scores for study 2 are calculated from more data points than in study 1. Both tasks also showed evidence of improvements in performance across the sessions (Table 6). Pairwise Wilcoxon signed rank tests (one-tailed) showed that these improvements were significant between sessions 1 and 2 (*Z* = 1.86, *p* < 0.05) and 2 and 3 (*Z* = 2.26, *p* < 0.05) for the Shopping List task. For the Squares task, there were also significant improvements between sessions 1 and 2 (*Z* = 3.06, *p* < 0.01), but the change between sessions 2 and 3 did not reach significance (*Z* = 1.31, *p* = 0.096), suggesting a plateauing of practice effects.

**Table 6:** Mean performance levels for the NANA cognitive tasks in each of the three testing periods of study 2

Task	Period 1	Period 2	Period 3	Friedman χ^2^ ^b^	Cross-session correlations (Kendall’s Tau values)		
	Mean of median RT (SD)	Mean of median RT (SD)	Mean of median RT (SD)				
					1 vs 2	2 vs 3	1 vs 3
SL (ms)	2613 (643)	2530 (547)	2445 (614)	14.10, *p* = .001	.73, *p* < .001	.77, *p* < .001	.76, *p* < .001
SqT (ms)	1032 (206)	975 (182)	967 (218)	25.99, *p* < .001	.66, *p* < .001	.72, *p* < .001	.63, *p* < .001

## Discussion

The results of study 2 show that the NANA cognitive tasks were feasible and valid measures of various domains of processing speed when administered in participants’ homes, without a researcher being present. This was true even when considering performance during 10-trial sessions of each task, showing that they are suitable for assessing patterns of cognitive function over micro-longitudinal timescales, and alongside other measures of function and behavior. As in study 1, performance on the Shopping List task was more strongly associated with standard cognitive tasks and participant age then performance on the Squares task was, suggesting that the former is better suited to tracking age-related patterns of cognitive functioning.

Performance on both of the NANA tasks showed evidence of some practice effects over the three testing periods, although these seemed to be plateauing out for the Squares task. In order to be able to more accurately detect changes in performance that are independent of practice effects, participants may therefore need to use the tasks for a longer period until asymptotic learning levels are reached (Blatter and Cajochen 2007).

## General discussion

Patterns of cognitive function over micro-longitudinal (hours–days) timescales are under-researched (Schmiedek et al. 2013), and yet are essential to our understanding of the mechanisms of cognitive aging (Lindenberger et al. 2007). To this end, we validated two simple cognitive tasks that can be administered in participants’ homes, without a researcher being present, as part of a broader battery of health and behavioral measures. Both tasks were shown to be usable and reliable and showed concurrent validity with a range of standard tests of cognition known to be sensitive to age- and health-related decline. The tasks therefore show promise as being informative measures of processing speed when administered without a researcher present.

Response times for both NANA tasks were correlated with performance on a range of standard cognitive tasks, although these correlations were stronger for the Shopping List task than the Squares task. The Shopping List task was designed to capture the principles of symbol substitution tasks, which are considered to largely depend on attention (Strauss et al. 2006), processing speed (Deary et al. 2010), and to be a marker of age- and health-related cognitive change (Lara et al. 2013). The strongest correlations for both NANA tasks were with the symbol substitution task, indicating that close operational correspondence was achieved. In addition, performance on the NANA tasks also correlated with measures of executive function (TMTB, Stroop, and digit span) and verbal episodic memory, consistent with the notion that processing speed underpins these higher-order abilities (Baltes and Lindenberger 1997; Tucker-Drob 2011). This demonstrates the advantage of measuring a more fundamental function, such as processing speed, in holistic assessment contexts such as the NANA system, as the increased efficiency of single tests minimizes the overall number of assessments that need to be administered.

The NANA tasks also show promise as indicators of more general changes in participants’ health and function. That is, although the predictive ability of the NANA cognitive tasks has not yet been assessed, they have shown convergent validity with other cognitive tasks that have been associated with a range of health outcomes. Importantly though, as the current studies were not designed to measure predictable patterns of cognitive change, such as those associated with diurnal variability (Baddeley et al. 1970) or experimentally induced physiological challenges (Balata et al. 2003; Somerfield et al. 2004), the ability of the tasks to reliably measure within-person changes in cognitive processing speed over micro-longitudinal timescales has yet to be established. The ability of the NANA tasks to reliably measure and predict changes in health and functional status therefore now needs to be formally tested in longitudinal studies.

Although the NANA tasks have been developed to assess micro-longitudinal patterns of cognitive processing speed in older adults, it is possible that they may also be useful for examining cognitive patterns in other populations of interest and over different timescales. For instance, the simple nature of the tasks, which were intentionally designed to be comprehensible without the need for a researcher, means that they may also be well suited for use with populations of children, or people with learning disabilities or cognitive impairment. Their use of response times rather than accuracy rates as a dependent variable also means that performance on the tasks will be less affected by ceiling effects. Again, further validation of the NANA cognitive tasks is now required in order to determine how well the tasks generalize to other populations.

There are some limitations of the current research. First, the participants who took part in both validation studies had relatively high levels of education and computer experience and showed little evidence of cognitive impairment. It is therefore unclear how well the tasks would perform and be tolerated by more diverse populations of older adults, or those with higher levels of cognitive impairment. Second, as the tasks involve language, imagers, and motor responses, they may be less well suited to older adults with language comprehension difficulties, or those with severe visual or motor impairments. Finally, the psychometric properties of the NANA cognitive tasks were only assessed over three occasions that were relatively close together. As practice effects (Strauss et al. 2006) and levels of acceptability (Palmier-Claus et al. 2013) can change over time period, further exploration of the performance of these tests over different timescales is now required.

In conclusion, the results of these studies show that the two computerized cognitive tasks developed for use in older people’s homes enable valid measures of cognitive processing speed to be collected without a researcher being present. Performance on the tasks was shown to be correlated with standard tasks of a range of cognitive function that are considered markers of healthy aging (Lara et al. 2013), providing convincing evidence that the NANA tasks will also demonstrate predictive validity of general health and functional ability. Further studies are now needed to determine the validity and usability of these tests when used in more diverse populations of older adults, and also to establish their ability to sensitively and reliably measure changes in cognitive function over various timescales.

## References

[CR1] American Psychiatric Association (2013) Diagnostic and Statistical Manual of Mental Disorders, 5th edn. Washington DC.

[CR2] Antunes HKM, Santos-Galduroz RF, Lemos VDA, Bueno OFA, Rzezak P, de Santana MG, De Melo MT (2015) The influence of physical exercise and leisure activity on neuropsychological functioning in older adults. Age 37. doi: 10.1007/s11357-015-9815-8. 10.1007/s11357-015-9815-8 PMC4501327 26169946

[CR3] Astell A, Hwang F, Brown L, Timon C, Maclean L, Smith T, Adlam T, Khadra H, Williams E (2014). Validation of the NANA (Novel Assessment of Nutrition and Ageing) touchscreen system for use at home by older adults. Exp Gerontol.

[CR4] Baddeley AD, Hatter JE, Scott D, Snashall A (1970). Memory and time of day. Q J Exp Psychol.

[CR5] Balata S, Damink SWMO, Ferguson K, Marshall I, Hayes PC, Deutz NEP, Williams R, Wardlaw J, Jalan R (2003). Induced hyperammonemia alters neuropsychology, brain MR spectroscopy and magnetization transfer in cirrhosis. Hepatology.

[CR6] Baltes PB, Lindenberger U (1997). Emergence of a powerful connection between sensory and cognitive functions across the adult life span: a new window to the study of cognitive aging?. Psychol Aging.

[CR7] Bird CM, Papadopoulou K, Ricciardelli P, Rossor MN, Cipolotti L (2004). Monitoring cognitive changes: psychometric properties of six cognitive tests. Brit J Clin Psychol.

[CR8] Blatter K, Cajochen C (2007). Circadian rhythms in cognitive performance: methodological constraints, protocols, theoretical underpinnings. Physiol Behav.

[CR9] Brown LJE, Adlam T, Hwang F, Khadra H, Maclean LM, Rudd B, Smith T, Timon C, Williams EA, Astell AJ (2016). Computerized self-administered measures of mood and appetite for older adults: the Novel Assessment of Nutrition and Ageing (NANA) toolkit. J Appl Gerontol.

[CR10] Brown LJE, Ferner HS, Robertson J, Mills NL, Pessotto R, Deary IJ, MacLullich AMJ (2011). Differential effects of delirium on fluid and crystallised abilities. Arch Gerotol Geriat.

[CR11] Capéraà P, Genest C (1993). Spearman’s *p* is larger than Kendall’s τ for positively dependent random variables. J Nonparametr Stat.

[CR12] Crawford JR, Deary IJ, Starr J, Whalley LJ (2001). The NART as an index of prior intellectual functioning: a retrospective validity study covering a 66-year interval. Psychol Med.

[CR13] Deary IJ, Johnson W, Starr JM (2010). Are processing speed tasks biomarkers of cognitive aging?. Psychol Aging.

[CR14] Field A (2013). Discovering statistics using IBM SPSS statistics.

[CR15] Folstein MF, Folstein SE, McHugh PR (1975). ‘Mini Mental State ’: a practical method for grading the cognitive state of patients for the clinician. J Psychiatr Res.

[CR16] Gamaldo AA, Allaire JC (2015). Daily fluctuations in everyday cognition. Is it meaningful?. J Aging Health.

[CR17] Graveson J, Bauermeister S, McKeown D, Bunce D (2015). Intraindividual reaction time variability, falls, and gait in old age: a systematic review. J Gerontol B-Psychol.

[CR18] Iverson G (1999). Interpretation of mini-mental state examination scores in community-dwelling elderly and geriatric neuropsychiatry patients. Int J Geriatr Psych.

[CR19] Jensen AR (1992). The importance of intraindividual variation in reaction time. Pers Indiv Differ.

[CR20] Lara J, Godfrey A, Evans E, Heaven B, Brown LJE, Barron E, Rochester L, Meyer T, Mathers JC (2013). Towards measurement of the Healthy Ageing Phenotype in lifestyle-based intervention studies. Maturitas.

[CR21] Lezak MD, Howieson DB, Bigler ED, Tranel D (2012). Neuropsychological assessment.

[CR22] Li S-C, Lindenberger U, Hommel B, Aschersleben G, Prinz W, Baltes P (2004). Transformations in the couplings among intellectual abilities and constituent cognitive processes across the life span. Psychol Sci.

[CR23] Lindenberger U, Li S-C, Lövdén M, Schmiedek F (2007). The Center for Lifespan Psychology at the Max Planck Institute for Human Development: overview of conceptual agenda and illustration of research activities. Int J Psychol.

[CR24] MacDonald SWS, Hultsch DF, Dixon RA (2003). Performance variability is related to change in cognition: evidence from the Victoria Longitudinal Study. Psychol Aging.

[CR25] May CP, Hasher L, Stoltzfus ER (1993). Optimal time of day and the magnitude of age differences in memory. Psychol Sci.

[CR26] McGurn B, Starr JM, Topfer JA, Pattie A, Whiteman MC, Lemmon HA, Whalley LJ, Deary IJ (2004). Pronunciation of irregular words is preserved in dementia, validating premorbid IQ estimation. Neurology.

[CR27] Mueller ST (2009) The PEBL manual (version 1.0). http://pebl.sourceforge.net. Accessed May 2010.

[CR28] Nelson HE (1982). National Adult Reading Test (NART): Test Manual.

[CR29] Palmier-Claus JE, Myin-Germeys I, Barkus E, Bentley L, Udachina A, Delespaul PAEG, Lewis SW, Dunn G (2011). Experience sampling research in individuals with mental illness: reflections and guidance. Acta Psychiat Scand.

[CR30] Palmier-Claus JE, Rogers A, Ainsworth J, Machin M, Barrowclough C, Laverty L, Barkus E, Kapur S, Wykes T, Lewis SW (2013). Integrating mobile-phone based assessment for psychosis into people’s everyday lives and clinical care: a qualitative study. BMC Psychiatry.

[CR31] Paradee CC, Rapport LJ, Hanks RA, Levy JA (2005). Circadian preference and cognitive functioning among rehabilitation inpatients. Clin Neuropsychol.

[CR32] Patterson CJS, Gauthier S, Bergman H, Cohen CA, Feightner JW, Feldman H, Hogan DB (1999). The recognition, assessment and management of dementing disorders: conclusions from the Canadian Consensus Conference on Dementia. Can Med Assoc J.

[CR33] Schmiedek F, Lövdén M, Lindenberger U (2013). Keeping it steady: older adults perform more consistently on cognitive tasks than younger adults. Psychol Sci.

[CR34] Sheik JI, Yesavage JA, Brink TL (1986). Geriatric Depression Scale (GDS): recent evidence and development of a shorter version. Clinical gerontology: a guide to assessment and intervention.

[CR35] Shipley BA, Der G, Taylor MD, Deary IJ (2007). Association between mortality and cognitive change over 7 years in a large representative sample of UK residents. Psychosom Med.

[CR36] Smith A (1982) Symbol Digit Modalities Test (SDMT). Manual (revised). Western Psychological Services, Los Angeles.

[CR37] Somerfield AJ, Deary IJ, Frier BM (2004). Acute hyperglycemia alters mood state and impairs cognitive performance in people with type 2 diabetes. Diabetes Care.

[CR38] Spreen O, Strauss E (1998). A compendium of neuropsychological tests: administration, norms, and commentary.

[CR39] Steinborn MB, Langner R, Flehmig HC, Huestegge L (2015). Everyday life cognitive instability predicts simple reaction time variability: analysis of reaction time distributions and delta plots. Appl Cognitive Psych.

[CR40] Strauss E, Sherman EMS, Spreen O (2006). A compendium of neuropsychological tests: administration, norms, and commentary.

[CR41] Stroop JR (1935). Studies of interference in serial verbal reactions. J Exp Psychol.

[CR42] Sullivan K (2005). Alternate forms of prose passages for the assessment of auditory-verbal memory. Arch Clin Neuropsych.

[CR43] Tierney MC, Naglie G, Upshur R, Moineddin R, Charles J, Jaakkimainen RL (2014). Feasibility and validity of the self-administered computerized assessment of mild cognitive impairment with older primary care patients. Alz Dis Assoc Dis.

[CR44] Timon CM, Astell AJ, Hwang F, Adlam TD, Smith T, Maclean L, Spurr D, Forster SE, Williams EA (2015). The validation of a computer-based food record for older adults: the Novel Assessment of Nutrition and Ageing (NANA) method. Brit J Nutr.

[CR45] Tucker-Drob EM (2011). Global and domain-specific changes in cognition throughout adulthood. Dev Psychol.

